# A lack of coordination between sister-chromatids segregation and cytokinesis in the oocytes of B6.Y^TIR^ (XY) sex-reversed female mice

**DOI:** 10.1038/s41598-017-00922-1

**Published:** 2017-04-19

**Authors:** Jia-Qiao Zhu, Seang Lin Tan, Teruko Taketo

**Affiliations:** 1 Department of Obstetrics and Gynecology, McGill University, Montreal, Quebec Canada; 2 Department of Surgery, McGill University, Montreal, Quebec Canada; 3 Department of Biology, McGill University, Montreal, Quebec Canada; 4 MUHC Reproductive Centre, Division of Reproductive Endocrinology and Infertility, McGill University, Montreal, Quebec Canada; 5 OriginElle Fertility Clinic and Women’s Health Centre, Montreal, Quebec Canada; 6 College of Veterinary Medicine, Yangzhou University, Yangzhou, Jiangsu P.R. China; 7 Jiangsu Co-innovation Center for Prevention and Control of Important Animal Infectious Diseases and Zoonoses, Yangzhou, Jiangsu P.R. China

## Abstract

The B6.Y^TIR^ (XY) mouse develops bilateral ovaries despite the expression of the testis-determining gene *Sry* during gonadal differentiation. We reported that the oocytes of the XY female are defective in their cytoplasm, resulting in a failure in the second meiotic division after activation or fertilization *in vitro*. However, the mechanism of meiotic failure or the cause of infertility remained to be clarified. In the present study, we obtained mature oocytes from XY females by superovulation and confirmed that these oocytes also fail in zygotic development. By using confocal microscopy 3D-analysis, we demonstrated that meiotic spindles were properly positioned and oriented in the MII-oocytes from XY females. After parthenogenic activation, fewer oocytes from XY females extruded the second polar body, and in those oocytes, sister-chromatids were often separated but neither set entered the second polar body. ARP2, F-actin, and ORC4, known to play roles in asymmetric meiotic division, were initially localized along the ooplasmic membrane and concentrated over the MII-spindle but lost their cortical polarity after activation while the sister-chromatids moved away from the oolemma in the oocytes from XY females. Our results indicate that the second polar body extrusion is uncoupled from the sister-chromatids separation in the oocytes from XY female mouse.

## Introduction

In mammalian development, the germ cells undergo sexual differentiation according to their gonadal environment, testis or ovary, which is determined by the presence or absence of the Y-linked *Sry* gene. Therefore, spermatogenesis and oogenesis take place in the presence of XY and XX sex chromosomes, respectively. When gonadal sex reversal occurs, however, the germ cell sex becomes discordant with the chromosomal sex. Both XX males and XY females in humans and XX males in mice are infertile while XY female mice show variable fertility dependent on the cause of sex reversal and genetic background (Reviewed by Taketo^1^). It must be noted that sex reversal results in functional gametes in some rodents and non-mammalian species such as *Akodon* and *Teleost* ^2–4^. Understanding the role of sex chromosomes in germ cell differentiation and functions would shed light on the evolution of sex chromosomes and meiotic mechanisms, which are unique to the germ cells.

In *Mus musculus*, the deletion of the Y chromosome region harboring *Sry* results in sex reversal (XY^*Tdym1*^ female) and subfertility on a mixed genetic background^5^. When most copies of *Rbmy* repeats on the Y chromosome are deleted, the *Sry* gene is repressed during gonadal differentiation and sex reversal ensues. These XY^*d1*^ females are nearly as fertile as XO females on the MF1 background^6^. These differences in XY female fertility have been attributed to the expression vs. repression of the Y-linked *Zfy2* gene on the MF1 background^7^. In the XY female on the B6 genetic background, named B6.Y^TIR^ or B6.Y^POS^, the *Sry* gene is intact and expressed during gonadal differentiation and yet fails to initiate testicular differentiation due to inefficient coordination with its target *Sox9* ^8–11^. These XY females are sterile except for one litter at an early backcross generation^8, 12^. Thus, the physical presence of the Y chromosome is compatible with female fertility, but the configuration or transcriptional activity of the Y chromosome appears to affect female fertility in the *Mus musculus* species.

Our laboratory has been particularly interested in the infertility of the B6.Y^TIR^ female mouse, which carries intact X and Y chromosomes. The B6.Y^TIR^ male mouse, which has developed uni- or bi-lateral testes, is fertile. Therefore, this mouse model provides a unique opportunity for comparing spermatogenesis and oogenesis with an identical chromosomal composition. We have previously reported that the germ cells in the B6.Y^TIR^ (named XY from herein) fetal ovary enter meiosis and go through the Meiotic Prophase I (MPI), but the X and Y chromosomes fail to pair at the pachytene stage, unlike the XY spermatocyte^12–14^. Subsequently, a greater number of XY oocytes are eliminated by the end of MPI, compared to XX oocytes. Nonetheless, a considerable number of XY oocytes survive to complete MPI and all stages of follicles can be found in the young XY ovary^12, 15^. When fully-grown oocytes are collected from XY ovaries and subjected to meiotic maturation *in vitro*, the unpaired X and Y chromosomes are segregated independently, producing some MII-oocytes carrying single X chromosomes^13, 16^. Nonetheless, very few oocytes reach the 2-cell-stage after fertilization, thus diminishing their chance for reproduction. This developmental incompetence of XY oocytes can be attributed to their defective cytoplasm; when the nuclei of XY oocytes have been transferred into enucleated XX oocytes, either at the GV- or MII-stage, the reconstructed oocytes generate healthy offspring after IVF and embryo transfer^17^.

In all our previous studies, XY oocytes were *in vitro* matured (IVM) for testing their developmental competence because the number of ovulated oocytes was too small to pursue further studies. However, IVM oocytes are known to be inferior to ovulated oocytes^18, 19^, and we cannot exclude the possibility that the cytoplasmic defects that we identified in the XY oocyte were exacerbated by the IVM conditions. In the present study, we succeeded to collect a reasonable number of oocytes from XY females by superovulation, allowing us to compare the developmental competence of oocytes matured *in vivo* with those matured *in vitro*. We then focused on the second meiotic progression and sister-chromatids segregation. Numerous studies have documented the second meiotic division in the mouse oocyte after fertilization at morphological and molecular levels, particularly the mechanism of release from the MII-arrest and meiotic progression initiated by the Ca^2+^-oscillation^20–26^. However, how the second meiotic division fails under physiological conditions is not well understood, compared with the first meiotic division, which involves the segregation of homologous chromosomes. As the frequency of aneuploidy at the second meiotic division is as high as that at the first meiotic division in human oocytes^27^, more studies are needed for understanding the mechanism of sister-chromatids segregation at the second meiotic division. Our XY female mouse provides a model for such a study. We examined the spindle conformation and sister-chromatids segregation in the mature oocytes following parthenogenic activation using time-lapse confocal microscopy imaging. Our results indicate that the second polar body extrusion is uncoupled from the sister-chromatids separation in the oocyte from XY female mouse.

## Results

### Ovulation of oocytes from XX and XY females with equine chorionic gonadotropin (eCG) injection

**Figure 1:**
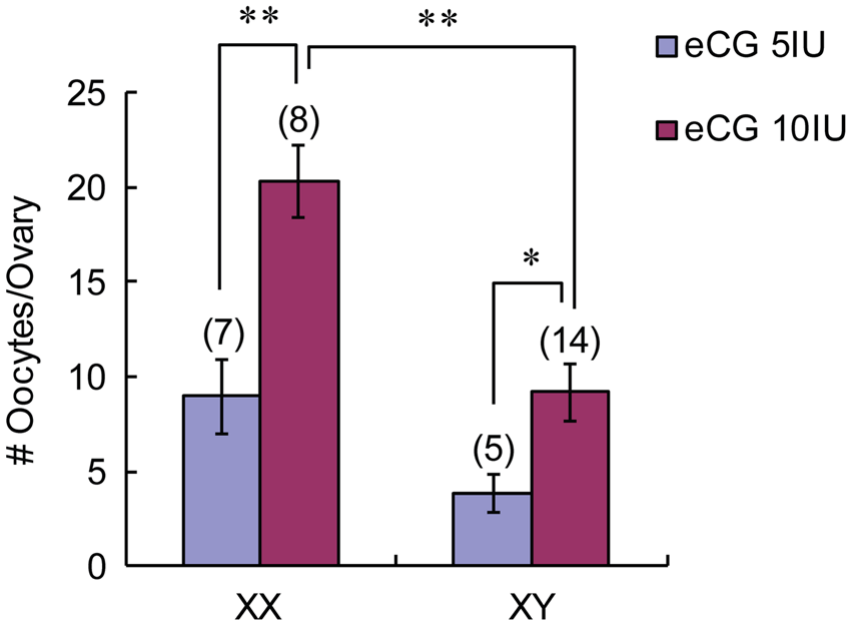
eCG-dosage dependent ovulation of oocytes from XX and XY females. The number of ovulated oocytes was larger after injection with 10 IU eCG than 5 IU eCG in both XX and XY females. The number of ovulated oocytes was also larger from XX females than XY females with 10 IU eCG. Data are presented as means ± SEM. The total number of females examined is given in parentheses at the top of each column. * and ** significant differences at p < 0.05 and 0.001, respectively.

We have previously reported that the B6.Y^TIR^ female at 25–35 days postpartum (dpp) ovulates very few oocytes (about 3/ovary) after injection with 5 IU equine chorionic gonadotropin (eCG) followed by 5 IU human chorionic gonadotropin (hCG) while the XX female ovulates about 10 oocytes/ovary^12^. This result prompted us to use IVM-oocytes from XY females for testing their developmental competence in subsequent studies^16, 17, 28^. However, it was possible that the defects in the oocytes of XY females may have been exacerbated by the IVM conditions. Accordingly, we revisited the possibility to obtain MII-oocytes from XY females by superovulation. As shown in Fig. 1, by increasing the dosage of eCG injection to 10 IU followed by 5 IU hCG, significantly larger numbers of oocytes were ovulated by both XX and XY females at 30–32 dpp (20.3 ± 1.9 and 9.1 ± 1.5/ovary, respectively) compared to 5 IU eCG (8.9 ± 2.0 and 3.8 ± 1.0/ovary, p < 0.001 and 0.05, respectively). At either eCG dosage, the number of ovulated oocytes from XY females was smaller than that from XX females although a significant difference was found only with 10 IU eCG. This finding allowed us to examine the developmental competence of oocytes matured *in vivo* (i.e., ovulation) from XY females and compare the results with those matured *in vitro*.

### Embryonic development of the ovulated oocytes from XX and XY females following IVF

**Figure 2:**
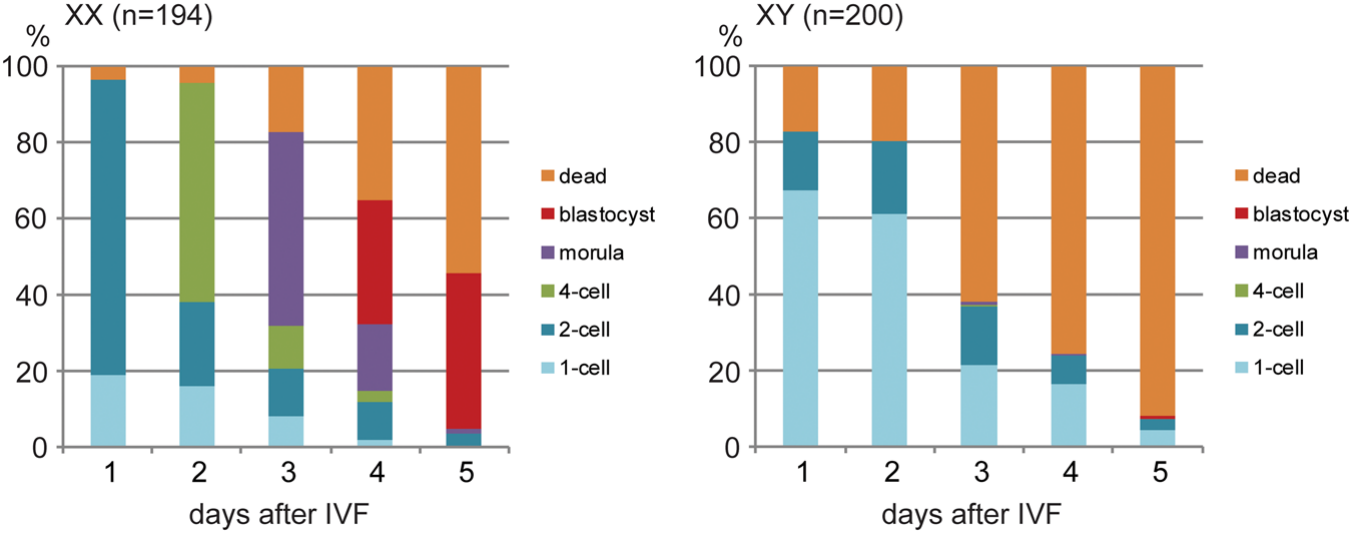
Failure in the embryonic development from the oocytes ovulated by XY females. Each column indicates the percentages of embryos at different stages at 1–5 days after IVF. “Dead” includes dead or fragmented embryos. The total number of oocytes examined is given in parentheses at the top of each graph.

We next tested the embryonic development from ovulated oocytes following IVF. The results are summarized in Fig. 2. From XX females, 77.3% (n = 194) of oocytes reached the 2-cell-stage at 1 day post-fertilization (dpf) and 41.2% reached the blastocyst-stage at 5 dpf. From XY females, 19.5% (n = 200) reached the 2-cell-stage while 61% were arrested at the 1-cell-stage at 1 dpf, and only one (0.5%) reached the blastocyst-stage while the rest were dead or fragmented at 5 dpf. These results indicate that the ovulated oocytes from XY females fail to initiate zygotic development, similarly to the IVM-oocytes^16, 17, 28^.

### Morphology and orientation of meiotic spindles in the MII-oocytes

**Figure 3:**
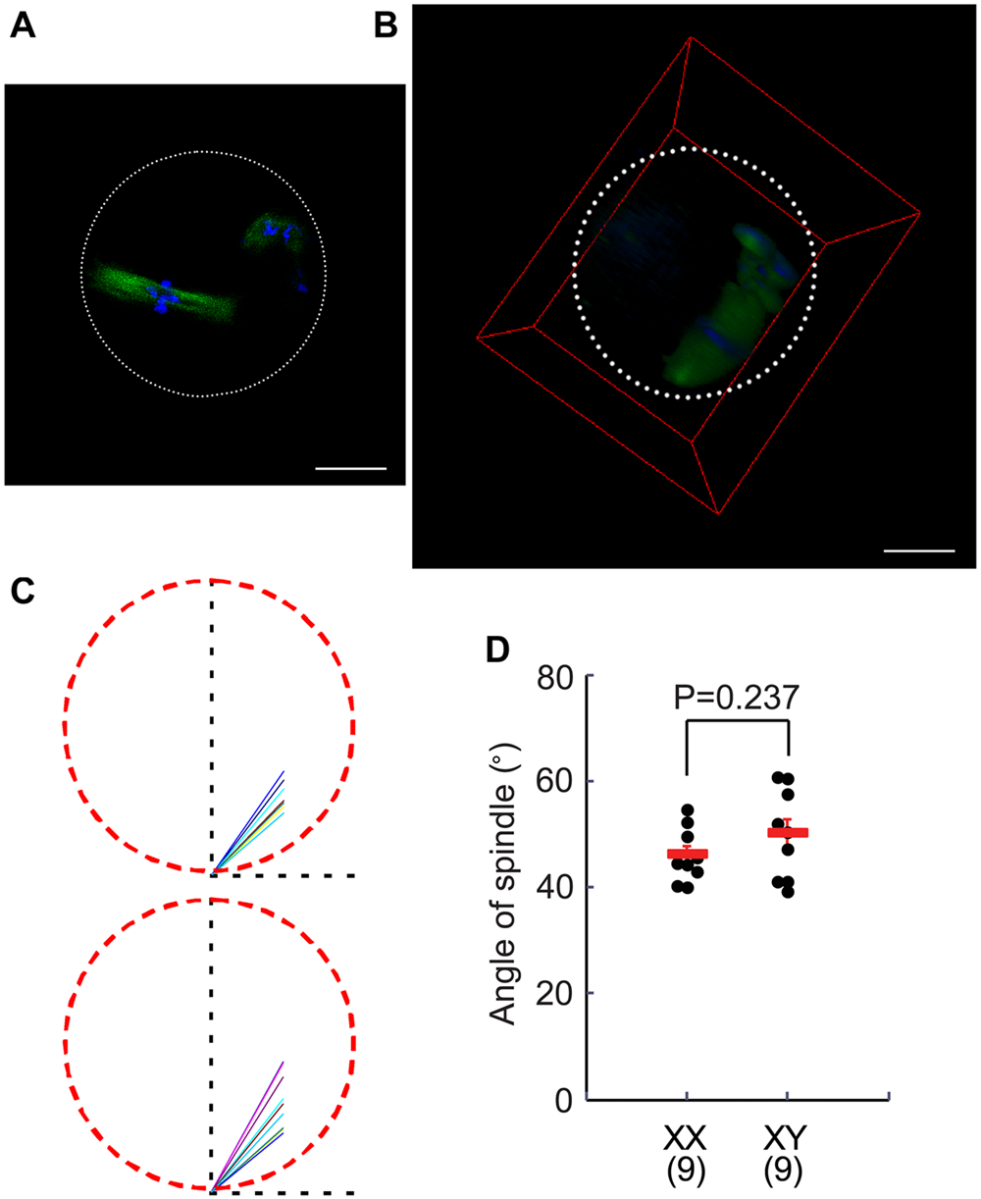
Normal orientation of MII-spindles in the IVM-oocytes from XY females. (**A**) 2D-image of a single z-section. An MII-oocyte from an XY female stained with anti-α-tubulin antibody (green) and DAPI (blue). The white broken circle indicates the position of zona pellucida. Bar: 20 µm. (**B**) 3D-image reconstructed from more than 40 z-sections at every 2.0 μm. The red box indicates the boundaries of 3D-image. The white broken circle indicates the position of zona pellucida. Bar: 20 µm. (The video of the 3D-image is given in Supplementary Fig. S1) (**C**) Summary of spindle orientation in the oocytes from XX and XY females. The red broken circle in each picture indicates the position of zona pellucida. The vertical axis was drawn to go through the center of each oocyte and the farther pole of the spindle. The spindle orientations in individual oocytes are shown in different colors. The angle of the spindle axis was measured from the horizontal axis in each oocyte. (**D**) Summery of spindle angles measured in the oocytes from XX and XY females. No difference is found between the two genotypes. Data are presented as means ± SEM. The total number of oocytes examined was given in parentheses at the bottom.

We have previously reported that meiotic spindles in the IVM oocytes from XY females are loosely organized and possibly in a wrong orientation^17, 29^. We had speculated that the improper spindle orientation might be responsible for the failure in the subsequent second meiotic division. However, in our previous study, we observed the spindle orientation in arbitrarily positioned oocytes on slides and could not confidently make the above conclusion. In the current study, we examined the meiotic spindles in the IVM-oocytes by confocal microscopy and constructed a 3D-model from more than 40 optical Z-sections at every 2.0 μm. Figure 3A shows an example of the 2D-image of a single Z-section obtained from an XY female, in which the microtubule spindle was loosely assembled and apparently oriented in a perpendicular position to the oolemma. However, the 3D-model of the same oocyte showed that the spindle was actually oriented in a parallel position to the oolemma (Fig. 3B), i.e., the two poles of spindle were in similar distances from the oolemma (Video Supplementary Fig. S1). The orientation of spindle was determined in each oocyte by drawing a vertical axis going through the center of oocyte and the farther pole of the spindle, and then the angle of spindle was measured from the horizontal axis (Fig. 3C). As the results are summarized in Fig. 3D, no difference was found in the spindle angle between the oocytes from XY females and those from XX females. These results demonstrate that the meiotic spindles are properly oriented in the IVM-oocytes from XY females.

### Frequency of aneuploidy in the MII-oocytes from XX and XY females

**Figure 4:**
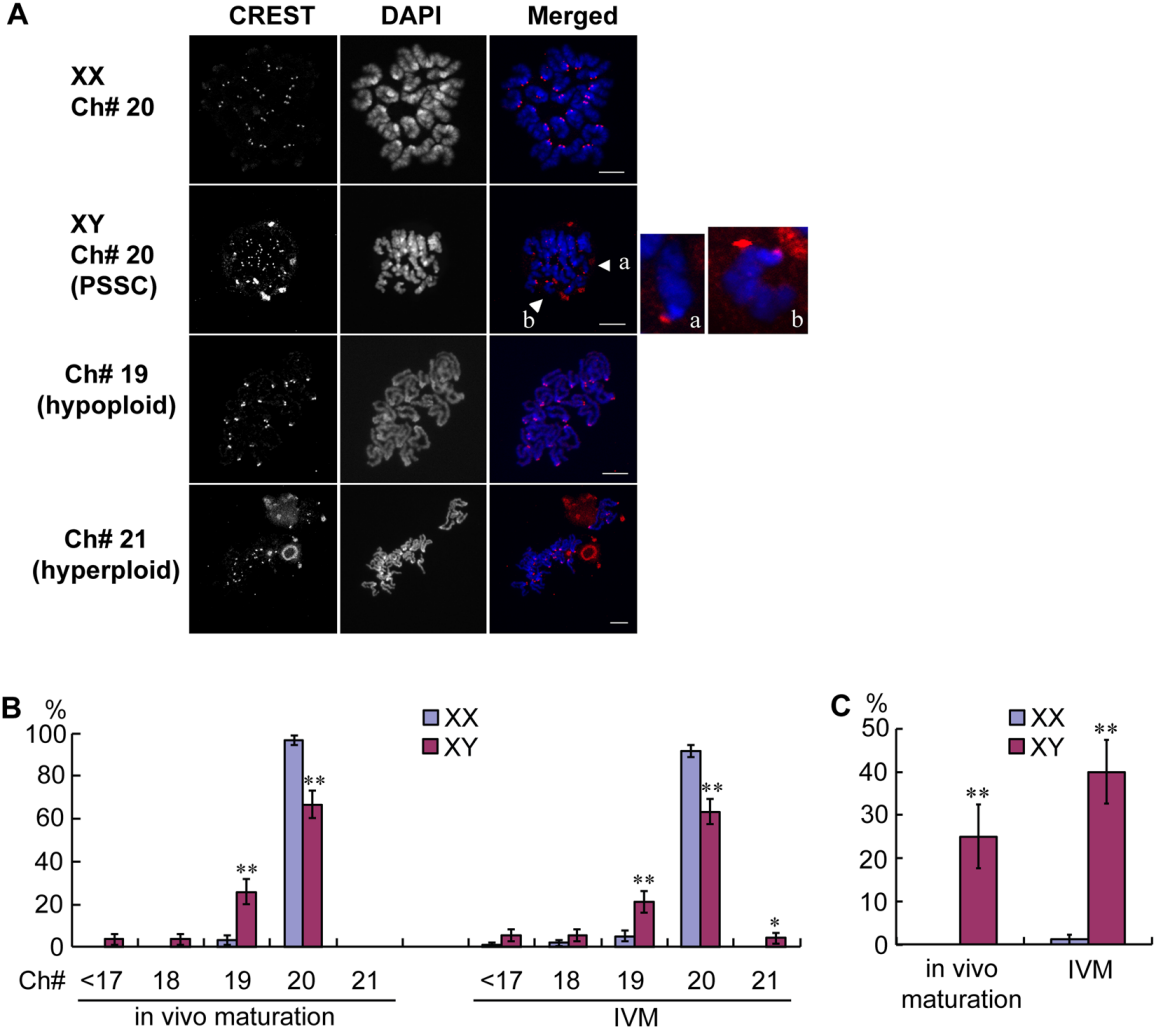
Higher frequency of aneuploidy in the MII-oocytes from XY females. (**A**) Examples of aneuploidy analyses. While almost all oocytes from XX females showed normal number (=20) of chromosomes, the oocytes from XY females showed variable numbers such as hypoploid (e.g. 19) or hyperploid (e.g., 21). The oocyte with the normal number of chromosomes often showed precocious separation of sister chromatids (PSSC). Bar: 10 µm. The areas around the chromatids indicated by (a) and (b) are magnified in the right panels. (**B**) Percentages of oocytes with normal (=20) and abnormal chromosome numbers. (**C**) Percentages of oocytes with PSSC. Data are shown as means ± SEM. * and ** significant differences at p < 0.05 and 0.01, respectively. The experiments were repeated at least three times each. For *in vivo* maturation, total 60 and 54 oocytes from XX and XY females, respectively, were examined. For IVM, total 95 and 73 oocytes from XX and XY females, respectively, were examined.

Aneuploidy is a major cause of embryonic development and spontaneous abortion^30–33^. A high incidence of aneuploidy and precocious separation of sister chromatids (PSSC) was reported for the IVM-oocytes from XO females, which lack the pairing partner for the single X chromosome^34–36^. We have also observed aneuploidy of sex chromosomes in the IVM-oocytes from XY females, which was inherited by the offspring that had been produced by nuclear transfer^16, 17^. In the current study, we asked whether the incidence of aneuploidy is lower in the ovulated oocytes. However, the results were largely consistent with those in the IVM-oocytes (Fig. 4). While almost all oocytes from XX females showed normal number (=20) of chromosomes, significantly higher percentages of the oocytes from XY females showed aneuploidy such as hypoploid (e.g. 19) or hyperploid (e.g. 21) (Fig. 4B). In addition, 25–40% of the euploid oocytes from XY females showed PSSC while almost none from XX females did so (Fig. 4C). Thus, the oocytes from XY females are inherently susceptible to chromosome segregation errors.

### Meiotic cell cycle progression and sister chromatids segregation in the MII-oocytes following parthenogenic activation

**Figure 5:**
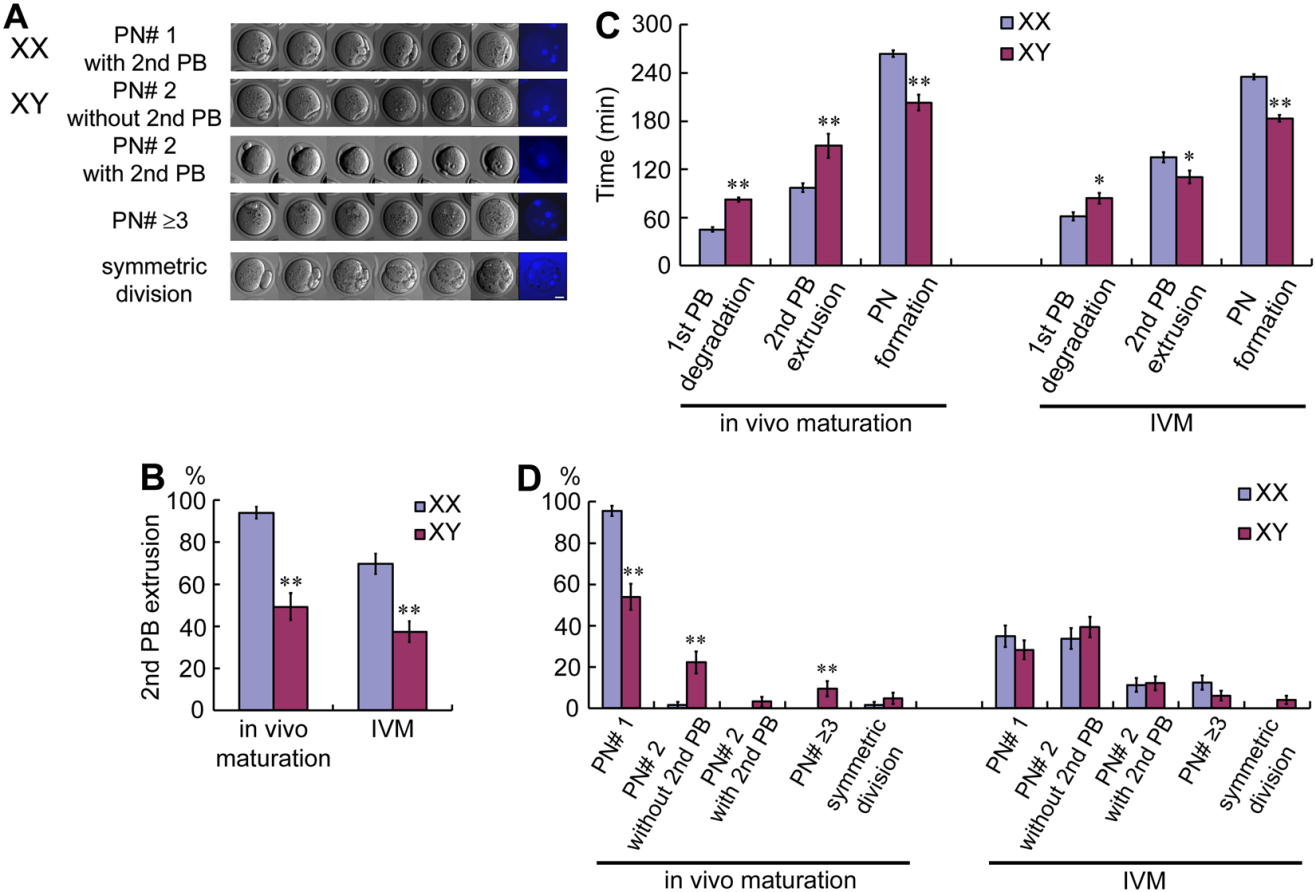
Meiotic progression and pronucleus formation in the MII-oocytes from XX and XY females following parthenogenic activation. (**A**) Time-lapse microscopy analysis of the second meiotic division. Chromosomes were stained with Hoechst 33342 (blue) at the end of time-lapse imaging. Five types of oocytes are shown; (1) a typical oocyte from an XX female with extrusion of the second polar body and single pronucleus formation. The oocytes from XY females are seen (2) without the second polar body extrusion and with two pronuclei formation, (3) with extrusion of the second polar body and two pronuclei formation, (4) without the second polar body extrusion and with multiple pronuclei formation, or (5) with symmetric cell division. Bar: 20 µm. (The time-lapse videos are given in Supplementary Fig. S2.1–S2.5) (**B**) Percentages of oocytes with the second polar body extrusion. (**C**) The time required for the oocyte to initiate the first polar body degradation, the second polar body extrusion, and pronuleus formation. (**D**) Percentages of oocytes that formed different numbers of pronuclei. Data are presented as mean ± SEM. * and **, significant differences between the oocytes from XX females and those from XY females at p < 0.05 and 0.001, respectively. The experiments were repeated at least three times each. For the *in vivo* maturation, total 67 and 63 oocytes from XX and XY females, respectively, were examined. For IVM, total 89 and 99 oocytes from XX and XY females, respectively, were examined.

We have previously reported that the GV-oocytes from XY females go through the first meiotic division *in vitro* similarly to those from XX females, but they fail to initiate zygotic development after IVF or parthenogenic activation^13, 16, 17, 28^. Furthermore, such oocytes formed multiple pronuclei, indicating sister-chromatids segregation errors^16^. However, multiple pronuclei are also frequently seen in the IVM-oocytes from XX females, suggesting a fundamental problem associated with IVM. In the present study, we compared the second meiotic division in the ovulated and IVM-oocytes from XX and XY females following parthenogenetic activation with 10 mM SrCl_2_. We used time-lapse imaging to monitor the progression of the second meiotic division, including the first polar body degeneration, the second polar body extrusion, and pronucleus formation (﻿Videos shown in Supplementary﻿ ﻿Fig. S2.1–S2.5). The typical results are shown in Fig. 5A. The XY oocyte often extruded an “empty” second polar body, which could be distinguished from the first polar body by time-lapse imaging (Video﻿ shown in Supplementary Fig. S2.3) and formed two or more pronuclei, which were visualized by Hoechst staining at the end of imaging (Fig. 5A). As summarized in Fig. 5B, 94.0% of the ovulated oocytes from XX females extruded the second polar body and almost all formed single pronuclei, whereas only 49.2% of the ovulated oocytes from XY females extruded the second polar body. Of these, 54.0% formed single pronuclei and the rest formed 2 or more pronuclei. In addition, 4.8% of oocytes divided symmetrically to resemble 2-cell-stage embryos. For comparison, 70.0% and 37.4% of the IVM-oocytes from XX and XY females, respectively, extruded the second polar body, and similar percentages of oocytes formed single or multiple pronuclei. Symmetric division was found only in the oocytes from XY females.

The kinetics of cell cycle progression were significantly altered in the oocytes, matured either *in vivo* or *in vitro*, from XY females compared to those from XX females (Fig. 5C). In the ovulated oocytes from XY females, degradation of the first polar body and extrusion of the second polar body, when it occurred, were delayed while formation of pronuclei was facilitated compared to those from XX females. In the IVM-oocytes from XY females, degradation of the first polar body was delayed while extrusion of the second polar body and formation of pronuclei were facilitated.

**Figure 6:**
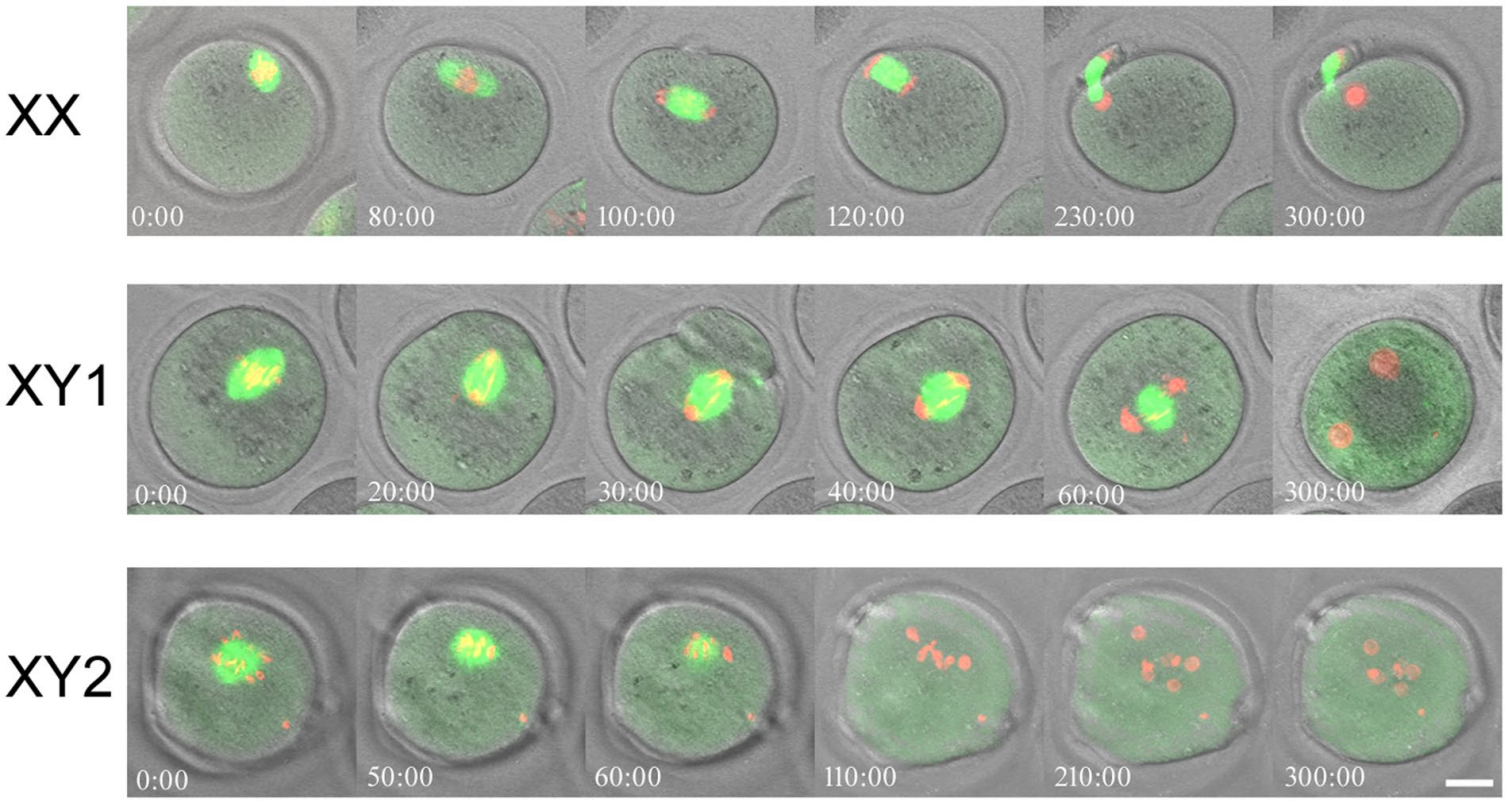
Spindle movement and sister chromatids segregation in the MII-oocytes from XX and XY females following parthenogenic activation. Meiotic spindles and chromosomes were visualized by injection of β5-tubulin-GFP and H2B-mCherry mRNAs into the GV-stage oocytes, followed by IVM. The images of oocytes from XY females were compressed from Z-stacks in order to capture the entire chromatids or pronuclei. Bar: 20 µm. (The time-lapse videos were given in Supplementary Fig. S3.1–S3.3.) The experiments were repeated at least three times each. Total 7 oocytes (1 or 2 oocytes at a time) were tested for each genotype.

To monitor the sister chromatids segregation in the oocytes after parthenogenetic activation in live, we microinjected β5-tubulin-GFP and H2B-mCherry mRNAs into the oocytes at the GV-stage, followed by IVM. The progression of the second meiotic division after activation was recorded by time-lapse imaging using the confocal microscopy. (Videos shown in Supplementary Fig. S3.1–S3.3.) Figure 6 shows that the MII-spindle in the oocytes from XX females behaved as expected; the spindle moved from the parallel to perpendicular position while sister chromatids were separated to the both spindle poles and a half of them were extruded into the second polar body within 300 min. By contrast, the MII-spindle in the oocyte from an XY female moved around without a clear destination and, although sister-chromatids were separated to the both spindle poles, all remained in the oocyte and formed multiple pronuclei (top), or the loosely aligned MII-chromosomes failed to segregate and formed multiple pronuclei without the second polar body extrusion (bottom).

In summary, the ovulated oocytes from XX females were much more efficient in the second meiotic division and sister-chromatids segregation compared to the IVM-oocytes. By comparison, the oocytes from XY females, which had been matured either *in vivo* or *in vitro*, exhibited difficulty in the second polar body extrusion. Even when the second polar body was extruded, neither set of sister-chromatids was expelled into the polar body.

### Localization of ORC4, ARP2, and F-actin in the MII-oocytes from XX and XY females

**Figure 7:**
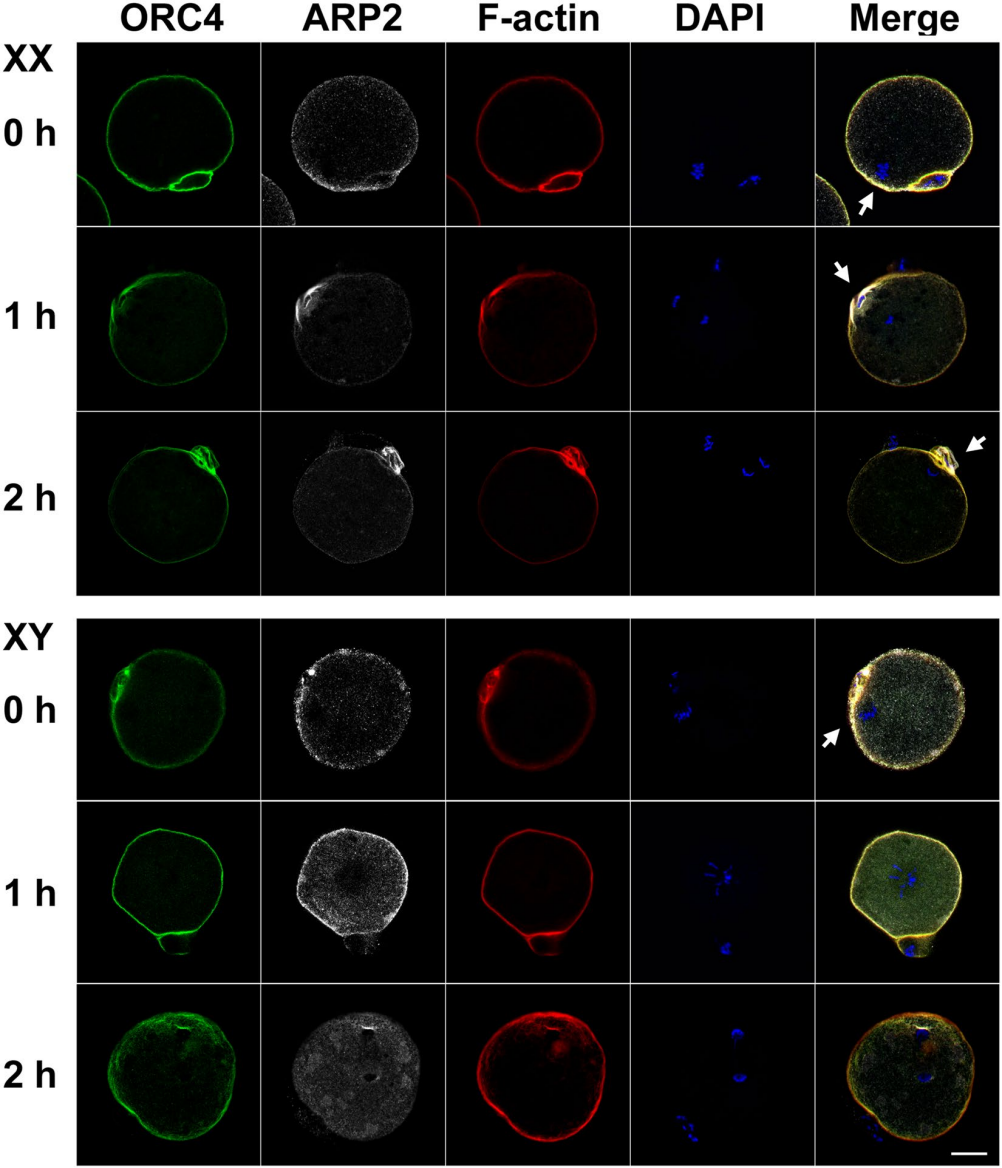
Loss of ORC4, ARP2 and F-actin polarization in the MII-oocytes from XY females after parthenogenic activation. ORC4, ARP2, and F-actin are localized along the oolemma and concentrated over the MII-spindle (arrows) in the ovulated-oocytes from both XX and XY females. ORC4 and F-actin are also concentrated but ARP2 is faint around the first polar body. After parthenogenic activation in the oocytes from XX females, all proteins are concentrated near the set of anaphase chromatids closer to oolemma (arrow) at 1 h and around the second polar body (arrow) at 2 h. In the oocyte from an XY female, by contrast, all proteins are localized evenly along the oolemma and, in addition, ARP2 has appeared in the ooplasm while the sister-chromatids are centered at 1 h after activation. In the oocyte at 2 h after activation, APR2 has disappeared from the oolemma entirely and distributed in the ooplasm while all ORC4, ARP2, and F-actin are concentrated around the set of anaphase chromatids near the oolemma. Bar: 20 µm. The experiments were repeated three times. Total 3, 10, and 10 oocytes of each genotype were examined at 0, 1, and 2 h, respectively.

To delineate the failure in the second meiotic division at the molecular level in the oocytes from XY females, we examined the localization of ORC4, ARP2, and F-actin, which may play direct roles in the cytokinesis during the second meiotic division^37–41^. The examples are shown in Fig. 7 (n = 3–10 each examined). In the MII-oocytes ovulated from either XX or XY females, all ORC4, ARP2 and F-actin were localized along the oolemma and concentrated over the MII-spindle. ORC4 and F-actin were also concentrated around the first polar body, but ARP2 was not. Following parthenogenic activation in the oocytes from XX females, all ORC4, ARP2 and F-actin were concentrated around the set of anaphase chromatids near the oolemma at 1 h and around the second polar body at 2 h. In the oocytes from XY females, by contrast, all ORC4, ARP2 and F-actin were seen evenly along the oolemma while the sister-chromatids moved away from the oolemma at 1 h after activation. In addition, ARP2 appeared in the ooplasm. At 2 h after activation, ARP2 had disappeared from the oolemma entirely and distributed in the ooplasm. ORC4 and F-actin were still localized along the oolemma and concentrated over the set of anaphase chromatids near the oolemma, but a cloud of immuno-staining was also seen in the nearby ooplasm. These results indicate that the cortical polarity was transiently lost and ARP2 was no longer properly localized on the oolemma after activation in the oocytes from XY females.

## Discussion

Our current results show that the oocytes from XY females went through the first meiotic division and assembled and positioned the MII-spindles normally during maturation *in vivo* or *in vitro*. Furthermore, the sister-chromatids were separated after activation while the second polar bodies were extruded in some oocytes from XY females. However, the anaphase chromatids often failed to be expelled into the second polar body, resulting in empty polar bodies and multiple pronuclei in the oocytes. We hypothesize that a loss of cortical polarity after activation impairs the coordination between the sister-chromatids separation and meiotic cytokinesis. Further studies are needed to identify the cytoplasmic components responsible for the loss of polarity in the oocytes from XY females.

For the research in reproduction, injection of eCG at 2–10 IU is commonly used to increase the number of meiotically competent oocytes collected from prepubertal or adult female mice^42–44^. However, there is a concern about the adverse effects of eCG treatment on the developmental competence of oocytes^44, 45^. Accordingly, we have been routinely using the lower effective dosage 5 IU of eCG for ovulation or collection of GV-oocytes for IVM^16, 46, 47^. With this dosage of eCG, an XX female produces total 20 oocytes, twice the number of natural ovulation, whereas an XY female produces maximum 6 oocytes at 35 dpp or none at 55 dpp^12^. In the current study, however, we found that injection of 10 IU eCG doubled the numbers of oocytes ovulated by both XX and XY females. These numbers are comparable with the numbers of MII-oocytes that can be obtained by IVM from each female^28^ and sufficient to examine their competence for embryonic development. Nonetheless, the number of ovulated oocytes from XY females was smaller than that from XX females at either eCG dosage. Such result may be explained by our previous finding that the number of follicles recruited into the growth phase is similar but fewer follicles reach the preovulatory phage in the XY ovary compared to the XX ovary^15^. The ovulated oocytes from XY females failed to develop beyond the 1- or 2-cell-stage, in agreement with the results with the IVM-oocytes^16, 28^. Therefore, the block of zygotic development is a major obstacle for the reproduction of XY females.

We have previously observed that MII-spindles are always positioned in the perpendicular orientation in the IVM-oocytes from XY females^17, 29^. However, as we arbitrarily mounted oocytes on histology slides, we could not exclude the possibility that the oocytes were positioned so that spindles appeared to be perpendicular but were actually parallel to the oolemma. Our current study supports the latter. We do not know why the oocytes from XY females settle on the slide in such a biased position while the oocytes from XX females appear in all orientations. We speculate that the distance of the MII-spindle from the oolemma may exhibit this effect. We did not examine the ovulated oocytes for this part of study as they are expected to be better than the IVM-oocytes.

Sex chromosomes rarely pair in the XY oocyte at the pachytene stage of MPI and segregate independently at the first meiotic division *in vitro* ^13, 16^. Furthermore, sex chromosome aneuploidy does not block the embryonic development from the XY oocyte nuclei transferred into the enucleated XX oocytes^17^. However, it was reported that the IVM-oocytes from XO females, which also lack the pairing partner for the single X chromosome, exhibit a high incidence of PSSC and chromosome lagging that is not limited to the X chromosome^34–36^. We confirmed that the incidence of PSSC was also high in the IVM-oocytes from XY females in the current study. However, such abnormal chromosome segregation can be exacerbated by the suboptimal IVM conditions. Therefore, we also tested the *in vivo* matured oocytes from XX and XY females, but obtained consistent results with the IVM-oocytes. Overall, aneuploidy was limited to the hypoproidy missing one chromosome and the hyperploidy with 21 chromosomes was even less frequent in our study. We conclude that the first meiotic division proceeds correctly other than sex chromosomes in the XY oocyte.

Our previous and current studies consistently show that the MII-oocytes from XY females are largely normal. These oocytes went through all meiotic stages after activation, including the first polar body degradation, sister-chromatids separation, the second polar body extrusion, and pronuclei formation. However, the coordination among these events was disturbed, reflecting in altered time lengths required for each event. Furthermore, despite the separation of sister-chromatids, neither set of the chromatids was expelled into the second polar body in most oocytes from XY females. For example, Video S2.3 shows that two pronuclei were formed before the extrusion of the second polar body. Video S3.2 shows that the MII- spindle moved to the center of the oocyte and formed two pronuclei there. For comparison, the MII-spindle in the oocyte from an XX female once moved away but returned to the oolemma and expelled a set of chromatids into the second polar body (Video S2.1). The movement of spindle in these videos was sluggish compared to a previous report^48^, probably because of the integration of exogenous histone and tubulin or the IVM conditions in our study. Moreover, the second meiotic division was monitored under the confocal microscope for 5 h, inevitably under suboptimal conditions. Nonetheless, the endpoint was distinct, corresponding to success and failure in the sister-chromatids segregation in most oocytes from XX females and those from XY females, respectively. Since variable cases were found after activation of oocytes from XY females (Fig. 5), a limited number of examples shown by videos may not represent all oocytes. Nonetheless, the results in most cases were consistent with a lack of coordination between sister-chromatids segregation and cytokinesis in the second meiotic division.

ARP2 is a component of the Arp2/3 complex (actin-related protein 2/3 complex) and co-localizes with actin in a Ran-GTPase-dependent manner^39, 49^. In the MII-oocyte, ARP2 generates a continuous actin flow from the cortical cap while it prevents the myosin-II-driven contraction of the cortical cap, maintaining a reverse cytoplasmic streaming^39^. As a net balance, the MII-chromosomes/spindle is maintained close to the oolemma. F-actin is essential for ARP2 localization and plays a key role in the asymmetric division and polar body extrusion^50, 51^. ORC4 is a subunit of the origin recognition complex (ORC) but may play a role in the oocyte outside of DNA replication; ORC4 is localized along the oolemma in the GV-oocyte and forms an ovoid structure surrounding the extruding anaphase chromosomes during both meiotic divisions^37, 52^. In our study, the MII-oocytes from XY females showed similar localization of ORC4, ARP2, and F-actin to those from XX females, suggesting that cortical localization of the MII-spindle and its downstream RanGTP gradient –ARP2/3 pathway was largely normal. However, the MII-spindle rapidly lost its cortical localization and moved away from the oolemma after activation in the oocytes from XY females. Interestingly, sister-chromatids were still capable of separating to the both poles while the oocyte underwent cytokinesis, either extruding the empty second polar body or dividing symmetrically. This manner of meiotic segregation failure differs from what has been described in the ARP2/3 deficient or aged oocytes^39, 53^. There must be a mechanism to maintain the meiotic spindle near the oolemma after activation, which is distinct from that at the MII-resting stage and defective in the oocytes from XY females. ORC4, ARP2, and F-actin were accumulated around the set of chromatids near the oolemma at 2 h after activation, suggesting that they have not lost their functions completely. However, ARP2 was no longer localized or associated with F-actin along the oolemma, and may have contributed to the lack of coordination between sister-chromatids segregation and cytokinesis. Our observation is somewhat similar but different from the deficiency in N-WASP, an activator of the Ran-ARP2 pathway, which allows normal MII-spindle assembly and positioning but inhibits the second polar body emission despite the initial sister-chromatids separation^54^.

A striking finding in the current study is the different response of the oocytes from XX and XY females to the two maturation conditions, *in vivo* and *in vitro*. On one hand, the ovulated oocytes from XX females did much better than IVM-oocytes, particularly after the second meiotic division; almost all ovulated oocytes extruded the second polar body and formed single pronuclei whereas only 35% of the IVM-oocytes did so. On the other hand, the oocytes from XY females performed similarly after maturation *in vivo* or *in vitro*. Although these results support the consistency in the defects of XY oocytes, they also suggest that the *in vivo* environment did not give the same benefit to the oocytes in XY females as those in XX females. We have previously reported a defect in the bidirectional communication between the oocyte and its surrounding granulosa/cumulus cells from the XY ovary in culture^29^. This lack of communication may persist *in vivo* and contribute to the ooplasmic defects. Alternatively, the XY oocyte may be already defective at the GV-stage and refractory to the maturation conditions.

## Methods

### Reagents

All chemicals were purchased from Sigma Chemical Co. (St. Louis, MO), unless otherwise stated.

### Mice

All animal experiments were conducted in accordance with the Guide to the Care and Use of Experimental Animals issued by the Canadian Council on Animal Care and with the approval by Animal Research Committee of McGill University. The B6.Y^TIR^ mouse was established by placing the Y chromosome from a variant of *Mus musculus domesticus* caught in Tirano, Italy (TIR) onto the C57BL/6 J (B6) genetic background by repeating backcrosses^9^. B6.Y^TIR^ male mice (N60-65 backcross generations) were crossed with B6 females (Jackson Laboratory, Bar Harbor, ME) to produce XY females and their XX littermates. The day of delivery was defined as 0 dpp. Upon weaning of pups at 20–25 dpp, their ear punches were taken and used for determining their genotypes by PCR amplification of the Y-linked *Zfy* gene as described previously^46^. All animals were maintained under specific-pathogen-free conditions with a humidity range of 30–60%, a temperature range 21–24 °C, a light cycle of 12 h light: 12 h darkness, and the food and water were provided *ad libitum*.

### Collection of *in vivo* matured oocytes by superovulation

Females at 28–30 dpp were injected intraperitoneally each with 5 or 10 IU eCG, followed by injection with 5 IU hCG 45–47 h later, and sacrificed 14–15 h later. The oocytes surrounded by cumulus cells, named cumulus-oocyte complexes (COCs), were flushed out from the oviduct and processed for either *in vitro* fertilization or microscopic examinations. For the latter, oocytes were denuded of cumulus cells by treatment with hyaluronidase (300 μg/ml) for 5 min at 37 °C and repeated pipetting through a narrow glass needle in MEM.

### *In vitro* fertilization (IVF) and embryonic development

Spermatozoa were collected from the caudal epididymis of (B6.DBA)F1 males at 10–12 weeks of age and capacitated in MEM-alpha (GIBCO, Life Technologies) supplemented with bovine serum albumin (BSA, 0.9%) under mineral oil at 37 °C for 60 min. COCs were transferred into an IVF medium (MEM-alpha supplemented with 0.4% BSA) containing diluted spermatozoa under oil and further incubated for 5 h at 37 °C with 5% CO_2_ in a humidified atmosphere. After washings in the modified KSOMaa medium (Millipore), the fertilized eggs were cultured in the fresh modified KSOMaa medium for up to 5 days.

### Collection of cumulus-oocyte complexes (COCs) and ***in vitro*** maturation (IVM) of oocytes

Females at 28–30 dpp were injected intraperitoneally each with 10 IU eCG and sacrificed 45–47 h later. Fully-grown GV-stage oocytes surrounded by cumulus cells were isolated by puncturing large preantral and antral follicles with a pair of 26-gauge needles in the MEM-alpha supplemented with fetal bovine serum (FBS, 5%) (GBICO, Life Technologies), streptomycin (50 μg/ml) and penicillin G potassium salt (75 μg/ml). Ten COCs in a group were cultured for 18 h in a pre-warmed 20 μl droplet of the maturation medium, MEM-alpha supplemented with FBS (5%), sodium pyruvate (25 μg/ml), FSH (300 ng/ml), streptomycin (50 μg/ml) and penicillin G potassium salt (75 μg/ml), under mineral oil at 37 °C with 5% CO_2_ in a humidified atmosphere.

### Confocal microscopy 3D-analysis of meiotic spindles

Denuded oocytes were fixed in a solution containing paraformaldehyde (2%, Electron Microscopy Sciences) and microtubule stabilizing buffer containing Pipes (100 mM, pH 6.9), MgCl_2_ (5 mM), EGTA (2.5 mM), Triton X-100 (0.5%), taxol (1 μM), aprotinin (10 IU/ml), and D_2_O (50%) for 30 min at room temperature. Then, oocytes were blocked in PBS containing BSA (3%) and Triton X-100 (0.1%) (named blocking solution) for 1 h at room temperature and incubated with mouse anti-α-tubulin-antibody-AlxaFluor488 (Life Technologies) (1:500) for 1 h. After three washes in PBS, oocytes were counterstained with DAPI (2 μg/ml, Behringer Manheim) in PBS. After washed three times, oocytes were transferred into PBS in a culture dish and examined under a confocal laser-scanning microscope (Zeiss LSM 780 META, Germany). To analyze the orientation of spindles, more than 40 optical sections of Z-series were obtained at 2.0 μm steps and the 3D-image was reconstructed by using the ImageJ software. The vertical axis was determined to go through the center of oocyte and the farther pole of spindle, and the angle of the long spindle axis from the horizontal axis was measured.

### Chromosome spreads and aneuploidy analysis in MII-oocytes

Metaphase chromosome numbers in the MII-oocytes were determined as previously described^55^ with minor modifications. Briefly, the zona pellucida was removed by incubation in Tyrode’s solution^56^, and the oocytes were dispersed in a solution containing paraformaldehyde (1%), Triton X-100 (0.15%) and DTT (3 mM) on Plus-charged histology slides (Thermo-Fisher Sci) and air dried. The slides were then immunofluorescence stained with anti-CREST antibody (ImmunoVision) (1:50), followed by goat anti-human IgG-Rhodamine (Jackson ImmunoResearch) (1:500), mounted with Prolong Antifade Mounting Medium containing DAPI (Molecular Probe, Life Technologies), and examined under the confocal laser-scanning microscope.

### Parthenogenetic activation of MII-oocytes with SrCl_2_ and live imaging

The MII-oocytes that had extruded the first polar body were transferred into a Ca^2+^-free M16 medium containing SrCl_2_ (10 mM). The cell cycle progression was monitored under an inverted microscope (Leica, DMIRB) equipped with a 37 °C incubator and 5% CO_2_ supply. At the end of monitoring, Hoechst 33342 was added into the incubation medium until the final concentration reached 1 μg/ml without disturbing the oocytes. The images were acquired 30 min later. IVM oocytes after activation were also monitored for up to 5 h by the confocal laser-scanning microscope equipped with a 37 °C incubator and 5% CO_2_ supply.

### Expression of exogenous β5-tubulin-GFP and H2B-mRFP1 in oocytes during IVM

The plasmid pRN3-β5-tubulin-GFP was kindly provided by Dr. Marie-Helene Verlhac (CIRB, Collège de France, Paris, France). The plasmid pCS-H2B-mRFP1 was obtained from Addgene (Cambridge, MA). These plasmids were linearized by *SfiI* and *NotI*, respectively, and their mRNAs were synthesized by *in vitro* transcription kit (Ambion). The purified mRNAs were microinjected into GV-stage oocytes by Narishige MMN-1 micromanipulators mounted on the Leica inverted microscope within 30 min. The mRNA-injected oocytes were further incubated in the MEM-alpha supplemented with milrinone (2.5 μM), BSA (3 mg/ml), streptomycin (50 μg/ml) and penicillin G potassium salt (75 μg/ml) for 2 h, followed by IVM as described above.

### Immunofluorescence staining of ORC4 and ARP2 in MII-oocytes

The MII-oocytes after activation for 0, 1 and 2 h were fixed as described above, and incubated with rabbit polyclonal anti-ORC4L antibody (AbCam) (1:200) and mouse monoclonal anti-ARP2 antibody (AbCam) (1:200) at room temperature for 1 h. After three washes, oocytes were incubated with goat-anti-mouse IgG-AlexaFluor647 (Invitrogen, Life Technologies) (1:500), goat-anti-rabbit IgG-FITC (Jackson ImmunoResearch) (1:500), and Rhodamine-Phalloidin-TRITC (5 IU/ml) at room temperature for 1 h. After three washes, oocytes were transferred into Prolong Antifade Mounting Medium containing DAPI on Plus-coated histology slides. Fluorescence signals were examined under the confocal laser-scanning microscope.

### Statistical analysis

All experiments were repeated at least three times. Data were presented as means ± SEM and statistically analyzed by ANOVA using the SPSS software.

## Electronic supplementary material

Supplementary information list

Fig S1

Fig S2.1

Fig S2.2

Fig S2.3

Fig S2.4

Fig S2.5

Fig S3.1

Fig S3.2

Fig S3.3
