# Supplementary material for: A lack of coordination between sister-chromatids segregation and cytokinesis in the oocytes of B6.YTIR (XY) sex-reversed female mice
Source: Sci Rep. 2017 Apr 19;7:960. doi: 10.1038/s41598-017-00922-1 (PMC5430445; doi:10.1038/s41598-017-00922-1)
Supplement: Supplementary file 1 — Supplementary information list [file 41598_2017_922_MOESM1_ESM.pdf]

SUPPLEMENTARY INFORMATION

A lack of coordination between sister-chromatids segregation and cytokinesis in the oocytes of B6.Y^{TIR} (XY) sex-reversed female mice

Jia-Qiao Zhu, Seang Lin Tan, and Teruko Taketo

Fig S1. Video of the 3D-image of a spindle in an MII-oocyte from an XY female.

Fig S2.1. Video of the meiotic cell-cycle progression from the MII-oocyte from an XX female after parthenogenic activation, which extruded the second polar body and formed single pronucleus.

Fig S2.2. Video of the meiotic cell-cycle progression from the MII-oocyte from an XY female after parthenogenic activation, which failed to extrude the second polar body and formed two pronuclei.

Fig S2.3. Video of the meiotic cell-cycle progression from the MII-oocyte from an XY female after parthenogenic activation, which excluded the second polar body but formed two pronuclei.

Fig S2.4. Video of the meiotic cell-cycle progression from the MII-oocyte from an XY female after parthenogenic activation, which failed to extrude the second polar body and formed multiple pronuclei.

Fig S2.5. Video of the meiotic cell-cycle progression from the MII-oocyte from an XY female after parthenogenic activation, which underwent symmetric cell division.

Fig S3.1. Video of spindle and sister-chromatids movement in the MII-oocyte from an XX female following parthenogenic activation.

Fig S3.2. Video of spindle and sister-chromatids movement in the MII-oocyte from an XY female following parthenogenic activation.

Fig S3.3. Video of spindle and sister-chromatids movement in the MII-oocyte from an XY female following parthenogenic activation.
